# Determinants of Quality of Life According to Cognitive Status in Parkinson’s Disease

**DOI:** 10.3389/fnagi.2020.00269

**Published:** 2020-08-20

**Authors:** Yun Fan, Xiaoniu Liang, Linlin Han, Yan Shen, Bo Shen, Chen Chen, Yimin Sun, Jian Wang, Yilin Tang

**Affiliations:** Department of Neurology and National Clinical Research Center for Aging and Medicine, Huashan Hospital, Fudan University, Shanghai, China

**Keywords:** Parkinson’s disease, mild cognitive impairment, dementia, quality of life, determinants

## Abstract

**Background:**

Quality of life (QoL) was worse in Parkinson’s disease patients with mild cognitive impairment (PD-MCI) or dementia (PDD) than PD patients with normal cognition (PD-NC). The aim of this study was to investigate and compare the potential heterogeneous determinants of QoL in PD patients with different cognitive statuses.

**Methods:**

We recruited 600 PD patients, including 185 PD-NC patients, 336 PD-MCI patients and 79 PDD patients, in this cross-sectional study. All patients completed the QoL assessment by the 39-item Parkinson’s Disease Questionnaire (PDQ-39), as well as clinical evaluations and neuropsychological tests. The determinants of the QoL were analyzed by multiple stepwise regression analysis.

**Results:**

QoL was more impaired across the three groups (PD-NC < PD-MCI < PDD). The Unified Parkinson’s Disease Rating Scale part III (UPDRS-III) score, Geriatric Depression Rating Scale (GDS) score and daily levodopa equivalent dose (LED) were independent variables of PDQ-39 in PD-NC patients. The GDS score, disease duration, UPDRS-III score, Epworth Sleepiness Score (ESS) and sex were independent variables of PDQ-39 in PD-MCI patients. The GDS score and disease duration were independent variables of PDQ-39 in PDD patients.

**Conclusion:**

The determinants of QoL in PD-NC, PD-MCI and PDD patients were heterogeneous. Motor function was considered to be the most crucial determinant for QoL in PD-NC, while depression was indicated to be the most vital determinant for PD-MCI and PDD. For QoL improvement, clinicians might need to focus more on motor function in PD-NC patients and on depression in PD-MCI and PDD patients.

## Introduction

Cognitive impairment is one of the most common NMS of PD, and up to 80% of PD patients ultimately suffer from dementia (PDD) (Hely et al., 2008). Mild cognitive impairment in PD (PD-MCI) represents a less severe cognitive deficit in patients (Petersen, 2011) and is considered a transition from unimpaired cognition to dementia (Petersen et al., 1999). Accruing evidence indicates that MCI is a predictor of dementia in PD (Hoogland et al., 2017).

It is vital to assess the QoL in PD patients, and QoL is considered to be a crucial outcome indicator in PD. PD is incurable at present, and the improvement or maintenance of QoL is an important objective of treatment and care in PD patients (Martinez-Martin, 2017). Accruing studies have reported that both motor symptoms and NMS make significant contributions to QoL in PD patients (Hinnell et al., 2012; Wu et al., 2014; Kuhlman et al., 2019). Cognitive impairment has also been shown to be correlated with poor QoL (Lawson et al., 2014, 2016).

Strong evidence revealed that the QoL was worse in both PD-MCI and PDD patients than PD patients with normal cognition (PD-NC) (Leroi et al., 2012; Lawson et al., 2014, 2016; van Uem et al., 2018). A longitudinal study reported that one of the most crucial determinants of QoL was baseline PD-MCI and that cognitive function made a much greater contribution to QoL in PD patients who developed dementia (Lawson et al., 2016). Studies from Italian and Russian cohorts both demonstrated that dementia was an independent determinant of QoL in PD (Winter et al., 2010, 2011). In addition, some studies found that some of the specific cognitive domains, such as impaired attention and memory, were associated with poor QoL in PD (Lawson et al., 2016; Vasconcellos et al., 2019). Most of the studies explored how cognitive impairment contributes to QoL in PD. However, there is a dearth of studies exploring the impact of clinical features on QoL across different cognitive statuses in patients with PD. To take more precise pharmacological and non-pharmacological interventions to improve or maintain QoL, clinicians ought to explore the clinical features and differences of QoL according to cognitive status in more detail. Therefore, we explored the potential different determinants of QoL in PD-NC, PD-MCI and PDD patients in this study.

## Materials and Methods

### Subjects

All subjects aged 50–80 years old were consecutively enrolled at Huashan Hospital, Fudan University from March 2011 to February 2019. Two neurologists specializing in movement disorders made the diagnosis of PD according to the United Kingdom Brain Bank criteria (Hughes et al., 1992). Cases with any history of stroke, epilepsy, encephalitis, traumatic brain injury, malignancies, cardiac events, or severe psychiatric illness were excluded from the study.

### Standard Protocol Approvals, Registrations, and Patient Consents

The study was approved by the Human Studies Institutional Review Board, Huashan Hospital, Fudan University. All patients provided their written informed consent in conformity to the Declaration of Helsinki to participate in our study.

### Clinical Assessments

Two physicians specializing in movement disorders performed the clinical and neuropsychological tests. Under the condition of anti-parkinsonian medications-off, the Unified Parkinson’s Disease Rating Scale-part III (UPDRS-III) was used to evaluate motor function. The GDS was used to evaluate depression (Ertan et al., 2005). The REM-sleep Behavior Disorder Screening Questionnaire (RBDSQ) was used to evaluate rapid-eye-movement (REM)-sleep behavior disorder (RBD) (Wang et al., 2015). The Epworth Sleepiness Scale (ESS) was used to evaluate another sleepiness problem, EDS (Chen et al., 2002). SSST-12 was used to evaluate olfaction function (Hummel et al., 2001). The dosage of anti-parkinsonian drugs was converted into a total daily levodopa equivalent dose (LED) for standardization of the medications data (Tomlinson et al., 2010).

In our study, QoL was measured by PDQ-39 which consists of 39 items, including eight subdomains: mobility (10 items), activity of daily living (6 items), emotional well-being (6 items), stigma (4 items), social support (3 items), cognition (4 items), communication (3 items), and bodily discomfort (3 items) (Tsang et al., 2002). It is the most commonly used and specific questionnaire for assessing QoL in PD patients. Each item of the PDQ-39 is scored on a 5-point Likert scale. In the current study, the PDQ-39 summary index (PDQ-39 SI) was standardized from the PDQ-39 original scores by dividing the scored points by the maximum possible points and then multiplying by 100. The PDQ-39 SI ranges from 0 to 100, with higher scores representing worse QoL. All the questionnaires used in the study were validated in Chinese version.

### Neuropsychological Tests and the Classification of PD-NC, PD-MCI and PDD

Patients who were taking regular anti-parkinsonian medications took the cognitive assessment. Global cognitive abilities were assessed in all patients using the MMSE (Katzman et al., 1988). A comprehensive battery of neuropsychological tests was used to examine five specific cognitive domains. The Symbol Digit Modalities Test (SDMT) (Sheridan et al., 2006; Goldman et al., 2015) and Trail Making Test A (TMT-A) (Zhao et al., 2013; Goldman et al., 2015) were used to evaluate attention and working memory. The Stroop Color-Word Test (CWT) (Steinberg et al., 2005) and Trail Making Test B (TMT-B) (Zhao et al., 2013; Goldman et al., 2015) were used to evaluate executive function. The Boston Naming Test (BNT) (Goldman et al., 2015) and Animal Fluency Test (AFT) (Lucas et al., 2005) were used to evaluate language. The Auditory Verbal Learning Test (AVLT) (Guo et al., 2009) and delayed recall of the Rey-Osterrieth Complex Figure Test (CFT-delay) (Caffarra et al., 2002) were used to evaluate memory. The Clock Drawing Test (CDT) (Guo et al., 2008) and copy task of the Rey-Osterrieth Complex Figure test (CFT) (Caffarra et al., 2002) were used to evaluate visuospatial function. The normative data and instructions for all the above neuropsychological tests are shown in Supplementary Table 1.

PDD was diagnosed based on the International Parkinson Movement Disorder Society (MDS) criteria (Dubois et al., 2007). PD-MCI was diagnosed based on the Level II criteria that the MDS Task Force published in 2012 (Litvan et al., 2012). A result was identified as abnormal if the score of a neuropsychological test was 1.5 SDs below the appropriate norms. Impairment on at least two neuropsychological tests, manifested by either two impaired tests in one cognitive domain or one impaired test in two different cognitive domains, was required for a diagnosis of PD-MCI. The remaining subjects who did not meet the criteria of dementia or MCI were identified as PD-NC.

### Statistical Analysis

Categorical variables were expressed as frequencies (%), and continuous variables were expressed as the mean ± standard deviation (SD) or median (25%, 75%). Among the three groups (PD-NC, PD-MCI and PDD), the Chi-squared test was used for comparing the categorical variables, and the Kruskal–Wallis test or one-way ANOVA test was used for comparing the continuous variables. For multiple comparison correction, Bonferroni correction was used for the Chi-squared test, and the Dwass, Steel, Critchlow-Fligner multiple comparison procedure (DSCF) was used for the Kruskal–Wallis test. The correlations between the clinical characteristics and PDQ-39 SI were analyzed by Spearman rank correlation analysis. Multiple stepwise regression, with age, sex, education, disease duration, LED, UPDRS-III score, GDS score, SSST-12 score, ESS and RBDSQ score entered, was applied to uncover the main determinants of QoL in PD-NC, PD-MCI and PDD patients. The R-squared (*R*^2^) index was used to determine the proportion of variance explained by the variables. Two-tailed *p-*values are presented. Differences were considered statistically significant at *P* < 0.05. The data analysis was conducted by SAS 9.4 (SAS Institute Inc., Cary, NC, United States).

## Results

### The Clinical Characteristics of PD-NC, PD-MCI and PDD Patients

In total, 635 patients aged 50–80 years who were diagnosed with PD were recruited. However, 35 subjects were excluded according to the specified study exclusion criteria, and the remaining 600 patients were selected. The clinical characteristics and cognitive profiles of the patients are shown in Table 1 and Supplementary Table 2. In the general information, there were no differences in sex, age, the dosage of levodopa and dopamine agonists and LED among the three groups. PD-NC patients had a greater number of education years in comparison with PD-MCI and PDD patients. PD-MCI patients had a longer disease duration than PD-NC patients, whereas there was no significant difference in comparison with PDD patients. Regarding motor symptoms, the PD-NC group showed lower UPDRS-III scores than the PD-MCI and PDD groups. The falls rate in PD-MCI and PDD patients was significantly higher than PD-NC patients. For NMS, in terms of depression, the GDS scores differed across the three groups (PD-NC < PD-MCI < PDD). In terms of sleep disorders, the ESS scores were remarkably distinct among the three groups. In terms of odor identification, the SSST-12 score in both the PD-NC and PD-MCI groups was significantly higher than that in the PDD group.

**TABLE 1:** Clinical characteristics of PD-NC, PD-MCI and PDD patients.

	**Total (*N* = 600)**	**PD-NC (*N* = 185)**	**PD-MCI (*N* = 336)**	**PDD (*N* = 79)**	*P*-value*	***P***-**value PD-NC vs. PD-MCI**	***P***-**value PD-NC vs. PDD**	***P***-**value PD-MCI vs. PDD**
Sex (M/F)	338/262	107/78	181/155	50/29	0.2788^#^	1.0000	1.0000	0.3879
Age (*y*)	61.93 ± 6.71	61.33 ± 6.68	62.06 ± 6.66	62.8 ± 6.93	0.2963	0.5118	0.2936	0.7328
Education (*y*)	10.67 ± 4.01	12.74 ± 2.9	9.58 ± 4.21	10.32 ± 3.6	<0.0001	<0.0001	<0.0001	0.5276
Disease duration (*y*)	4.42 (1.67, 9.67)	3.08 (1.50, 7.17)	4.96 (2.00, 10.71)	5.17 (1.67, 10.42)	0.0054	0.0043	0.1331	0.9512
LED (mg/day)	525.20 (500.00, 825.60)	450.00 (300.00, 750.40)	537.50 (300.00, 825.60)	600.00 (331.55, 1000.50)	0.1308	0.2188	0.1987	0.7170
Levodopa (mg/day)	400.00 (225.60,600.79)	300.40 (225.59,600.39)	400.00 (238.00,600.00)	500.00 (300.00,750.00)	0.2897	0.7353	0.2689	0.4708
Dopamine agonists (mg/day)	75.00 (50.00,150.00)	75.00 (50.00,125.00)	75.00 (50.00,150.00)	100.00 (50.00,150.00)	0.3695	0.8299	0.3632	0.4874
UPDRS-III score	34.93 ± 15.79	28.39 ± 14.12	37.55 ± 15.26	41.6 ± 16.99	<0.0001	<0.0001	<0.0001	0.2466
Fall, yes, *n* (%)	111 (18.50)	20 (10.81)	69 (20.54)	22 (27.85)	0.0014^#^	0.0082	0.0028	0.2200
GDS score	12.02 ± 7.41	10.26 ± 7.46	12.35 ± 7.15	14.84 ± 7.38	<0.0001	0.0018	<0.0001	0.0254
ESS	6.49 ± 4.83	5.90 ± 4.53	6.49 ± 4.61	7.90 ± 6.02	0.0487	0.2547	0.0566	0.2929
RBDSQ score	4.57 ± 3.15	4.24 ± 2.96	4.68 ± 3.21	4.97 ± 3.35	0.2779	0.4068	0.3302	0.8285
SSST-12 score	4.78 ± 2.38	5.17 ± 2.54	4.81 ± 2.22	3.73 ± 2.31	0.0002	0.2703	0.0002	0.0016
MMSE	26.44 ± 3.32	28.21 ± 1.58	26.79 ± 2.28	20.76 ± 3.98	<0.0001	<0.0001	<0.0001	<0.0001

ESS, Epworth Sleepiness Score; GDS, Geriatric Depression Rating Scale; LED, levodopa-equivalent daily dose; MMSE, Mini Mental State Examination; PDD, Parkinson’s disease with dementia; PD-MCI, Parkinson’s disease with mild cognitive impairment; PD-NC, Parkinson’s disease with normal cognition; RBDSQ, Rapid-Eye-Movement Sleep Behavior Disorder Screening Questionnaire; SSST-12, Sniffin’ Sticks screening 12 test; UPDRS-III, Unified Parkinson’s Disease Rating Scale part III. The data of disease duration, levodopa, dopamine agonists and levodopa equivalent dose are presented as median (25%, 75%), and the other continuous data are presented as mean ± SD. *Comparison among the three groups with PD-NC, PD-MCI, and PDD. ^#^The categorical variables were compared among the three groups by Chi-squared test. The continuous variables were compared among the three groups by Kruskal–Wallis test.

### QoL Assessment in PD-NC, PD-MCI and PDD Patients

**FIGURE 1:**
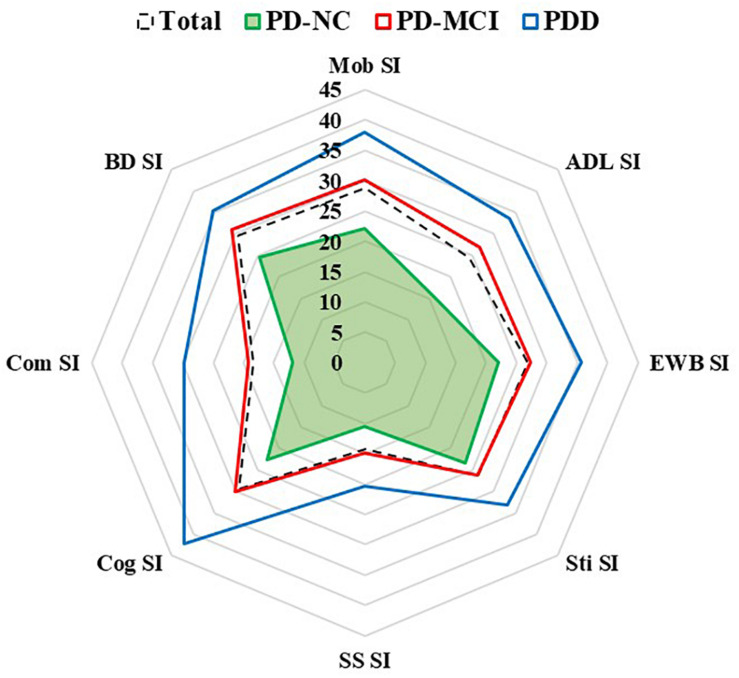
Subdomains of quality of life in PD-NC, PD-MCI and PDD patients. Quality of life was assessed by eight PDQ-39 subscales. ADL, activities of daily living; BD, bodily discomfort; Cog, cognition; Com, communication; EWB, emotional well-being; Mob, mobility; PDD, Parkinson’s disease with dementia; PD-MCI, Parkinson’s disease with mild cognitive impairment; PD-NC, Parkinson’s disease with normal cognition; SI, summary index; SS, social support; Sti, stigma.

The results of QoL assessed by the PDQ-39 are shown in Table 2 and Figure 1. The QoL was more impaired across the three groups (PD-NC < PD-MCI < PDD). The most affected subdomain of the PDQ-39 was bodily discomfort (29.63 ± 23.55), followed by cognition (29.40 ± 21.38) and mobility (28.68 ± 25.96) in all PD patients. Furthermore, the most impaired subdomains were bodily discomfort (24.59 ± 20.57) and stigma (23.28 ± 24.98) in PD-NC patients, while it was bodily discomfort (31.05 ± 23.51) and cognition (30.15 ± 19.83) in PD-MCI patients, and cognition (42.09 ± 26.34) and mobility (38.04 ± 28.79) in PDD patients.

**TABLE 2:** Quality of life assessment in PD-NC, PD-MCI and PDD patients.

	**Total (*N* = 600)**	**PD-NC (*N* = 185)**	**PD-MCI (N-336)**	**PDD (*N* = 79)**	*P*-value*	***P*-value PD-NC vs PD-MCI**	***P*-value PD-NC vs PDD**	***P*-value PD-MCI vs PDD**
PDQ-39 SI	25.50 ± 18.05	19.78 ± 15.69	26.60 ± 17.48	34.20 ± 21.20	<0.0001	<0.0001	<0.0001	0.0125
Mobility SI	28.68 ± 25.96	22.19 ± 23.27	30.07 ± 25.87	38.04 ± 28.79	<0.0001	0.0007	<0.0001	0.0688
Activity of daily living SI	24.32 ± 25.15	15.78 ± 20.45	26.86 ± 25.11	33.66 ± 29.63	<0.0001	<0.0001	<0.0001	0.263
Emotional well-beings SI	26.75 ± 23.08	22.07 ± 20.41	27.21 ± 23.16	35.76 ± 25.91	0.0001	0.0499	<0.0001	0.0155
Stigma SI	26.26 ± 26.24	23.28 ± 24.98	26.32 ± 26.09	33.12 ± 28.80	0.0399	0.3822	0.0333	0.1782
Social support SI	14.26 ± 23.69	10.54 ± 19.64	14.92 ± 24.40	20.35 ± 27.92	0.0052	0.1339	0.0034	0.1147
Cognitions SI	29.40 ± 21.38	22.64 ± 19.02	30.15 ± 19.83	42.09 ± 26.34	<0.0001	<0.0001	<0.0001	0.0008
Communication SI	18.33 ± 21.93	11.96 ± 17.97	19.22 ± 21.63	29.76 ± 26.40	<0.0001	<0.0001	<0.0001	0.0025
Bodily discomfort SI	29.63 ± 23.55	24.59 ± 20.57	31.05 ± 23.51	35.47 ± 27.99	0.0035	0.0103	0.0163	0.5891

PDD, Parkinson’s disease with dementia; PD-MCI, Parkinson’s disease with mild cognitive impairment; PD-NC, Parkinson’s disease with normal cognition; PDQ-39, 39-item Parkinson’s disease questionnaire; SI, summary index. *Comparison among the three groups with PD-NC, PD-MCI, and PDD.

### Correlation Analysis of Clinical Characteristics and QoL in PD-NC, PD-MCI and PDD Patients

Correlation analysis between clinical characteristics and PDQ-39 SI was performed in all PD patients (Table 3). GDS score, UPDRS-III score, disease duration, LED, ESS and RBDSQ score were positively associated with PDQ-39 SI, while education and SSST-12 score were negatively correlated with PDQ-39 SI.

**TABLE 3:** Correlation analysis of clinical characteristics and PDQ-39 SI in PD-NC, PD-MCI and PDD patients.

	**PDQ-39**							
	**Total**		**PD-NC**		**PD-MCI**		**PDD**	
	***r*_*s*_**	***P*-value**	***r*_*s*_**	***P*-value**	***r*_*s*_**	***P*-value**	***r*_*s*_**	***P*-value**
GDS score	0.6597	<0.0001	0.6464	<0.0001	0.6080	<0.0001	0.7478	<0.0001
UPDRS-III score	0.6138	<0.0001	0.5681	<0.0001	0.5500	<0.0001	0.6468	<0.0001
Disease duration (y)	0.4929	<0.0001	0.4369	<0.0001	0.4877	<0.0001	0.5464	<0.0001
LED (mg/day)	0.4371	<0.0001	0.5407	<0.0001	0.3809	<0.0001	0.3568	0.0028
ESS	0.3502	<0.0001	0.3302	<0.0001	0.3110	<0.0001	0.4601	<0.0001
RBDSQ score	0.2483	<0.0001	0.2133	0.0044	0.2454	<0.0001	0.3109	0.0105
Age (y)	0.0184	0.6526	−0.0790	0.2853	0.0657	0.2299	−0.0031	0.9785
SSST-12 score	−0.0930	0.0348	−0.0843	0.2848	−0.0434	0.4684	−0.0465	0.7025
Education (y)	−0.1621	<0.0001	−0.1188	0.1082	−0.0324	0.5616	−0.2963	0.0080

*ESS, Epworth Sleepiness Score; GDS, Geriatric Depression Rating Scale; LED, levodopa-equivalent daily dose; PDD, Parkinson’s disease with dementia; PD-MCI, Parkinson’s disease with mild cognitive impairment; PD-NC, Parkinson’s disease with normal cognition; PDQ-39, 39-item Parkinson’s disease questionnaire; RBDSQ, Rapid-Eye-Movement Sleep Behavior Disorder Screening Questionnaire; SSST-12, Sniffin’ Sticks screening 12 test score; UPDRS- III, Unified Parkinson’s Disease Rating Scale part III.*

Then, we explored the impact of clinical characteristics on QoL in PD patients with different cognitive states. In PD-NC and PD-MCI patients, GDS score, UPDRS-III score, disease duration, LED, ESS and RBDSQ score were positively correlated with PDQ-39 SI. In PDD patients, GDS score, UPDRS-III score, disease duration, ESS, LED and RBDSQ score were positively correlated with PDQ-39 SI, while education was negatively correlated with PDQ-39 SI.

### Determinants of QoL in PD-NC, PD-MCI and PDD Patients

To reveal the determinants of QoL in PD patients, we conducted a multiple stepwise analysis with age, sex, education, disease duration, LED, UPDRS-III score, GDS score, SSST-12 score, ESS and RBDSQ score entered (Table 4). In all the PD patients, the most severe determinant of the PDQ-39 was GDS score (*R*^2^ = 0.41, β = 1.05, *P* < 0.0001), followed by UPDRS-III score (*R*^2^ = 0.13, β = 0.35, *P* < 0.0001), ESS (*R*^2^ = 0.02, β = 0.49, *P* = 0.0003), female (*R*^2^ = 0.01, β = 4.20, *P* = 0.0006) and disease duration (*R*^2^ = 0.01, β = 0.38, *P* < 0.0046).

**TABLE 4:** Determinants of life quality in PD-NC, PD-MCI and PDD patients.

	***R*^2^**	**β**	**95% CI**	***P*-value**
**Total (*R*^2^ = 0.617)**				
GDS score	0.4113	1.052	(0.888, 1.217)	<0.0001
UPDRS-III score	0.1295	0.355	(0.268, 0.441)	<0.0001
LED (mg/day)	0.0391	0.003	(−0.000, 0.007)	0.0525
ESS	0.0151	0.488	(0.228, 0.749)	0.0003
Gender (male)	0.0092	−4.200	(−6.594, −1.805)	0.0006
Disease duration (*y*)	0.007	0.383	(0.119, 0.648)	0.0046
RBDSQ score	0.0056	0.383	(−0.003, 0.768)	0.0518
**PD-NC (*R*^2^ = 0.646)**				
UPDRS-III score	0.3575	0.366	(0.225, 0.506)	<0.0001
GDS score	0.2067	0.892	(0.633, 1.151)	<0.0001
LED (mg/day)	0.0649	0.010	(0.005, 0.015)	0.0002
SSST-12 score	0.0091	−0.488	(−1.193, 0.217)	0.1728
Gender (male)	0.0076	−1.853	(−5.634, 1.928)	0.3332
**PD-MCI (*R*^2^ = 0.536)**				
GDS score	0.3459	1.050	(0.833, 1.267)	<0.0001
Disease duration (*y*)	0.1092	0.488	(0.222, 0.753)	0.0004
UPDRS-III score	0.0499	0.381	(0.271, 0.490)	<0.0001
ESS	0.0158	0.440	(0.107, 0.773)	0.0099
Gender (male)	0.0077	−3.377	(−6.341, −0.414)	0.0257
RBDSQ score	0.0071	0.358	(−0.110, 0.825)	0.1331
**PDD (*R*^2^ = 0.758)**				
GDS score	0.5096	1.809	(1.371, 2.247)	<0.0001
ESS	0.1305	0.495	(−0.097, 1.088)	0.0998
Age (*y*)	0.0565	0.323	(−0.131, 0.778)	0.1599
Disease duration (*y*)	0.0441	0.829	(0.192, 1.467)	0.0116
RBDSQ score	0.0177	0.900	(−0.130,1.931)	0.0857

*β, standardized beta coefficient; CI, confidence interval; ESS, Epworth Sleepiness Score; GDS, Geriatric Depression Rating Scale; LED, levodopa-equivalent daily dose; PDD, Parkinson’s disease with dementia; PD-MCI, Parkinson’s disease with mild cognitive impairment; PD-NC, Parkinson’s disease with normal cognition; RBDSQ, Rapid-Eye-Movement Sleep Behavior Disorder Screening Questionnaire; SSST-12, Sniffin’ Sticks screening 12 test; UPDRS-III, Unified Parkinson’s Disease Rating Scale part III.*

Then, we explored the determinants of QoL across different cognitive statuses in PD. In PD-NC patients, the most important determinant of QoL was UPDRS-III score (*R*^2^ = 0.36, β = 0.36, *P* < 0.0001), followed by GDS score (*R*^2^ = 0.21, β = 0.89, *P* < 0.0001) and LED (*R*^2^ = 0.07, β = 0.01, *P* = 0.0002). In PD-MCI patients, the most vital determinant of QoL was GDS score (*R*^2^ = 0.35, β = 1.05, *P* < 0.0001), followed by disease duration (*R*^2^ = 0.11, β = 0.49, *P* = 0.0004), UPDRS-III score (*R*^2^ = 0.05, β = 0.38, *P* < 0.0001), ESS (*R*^2^ = 0.02, β = 0.44, *P* = 0.0099) and female (*R*^2^ = 0.01, β = 3.38, *P* = 0.0257). In PDD patients, the most vital determinant of QoL was also GDS score (*R*^2^ = 0.51, β = 1.8, *P* < 0.0001), followed by disease duration (*R*^2^ = 0.04, β = 0.83, *P* = 0.0116).

## Discussion

The present study demonstrated that QoL was remarkably worse in PD-MCI and PDD patients than in PD-NC patients. To our knowledge, this study has revealed the heterogeneous determinants of QoL across different cognitive statuses in PD for the first time. The UPDRS-III score was considered the most important determinant for PD-NC, while depression was proven to be the major determinant for PD-MCI and PDD. These findings may prompt clinicians to focus on specific factors for improving QoL according to the cognitive status in PD.

Consistent with previous studies (Lawson et al., 2014, 2016; van Uem et al., 2018), our study found that the more severe the cognitive impairment, the higher the PDQ-39 score. QoL was worse across the three groups: PD-NC < PD-MCI < PDD. However, a United Kingdom study reported that QoL was similar between PD-NC and PD-MCI groups, although QoL was significantly worse in PDD patients (Leroi et al., 2012). The above discrepancy could be attributed to the fact that the Level I criteria were used for defining the “possible” PD-MCI in this study. Here, PD-MCI was diagnosed according to MDS level II category guidelines, which are considered more stringent criteria. As such, patients with more impaired cognitive function were included in the PD-MCI group. Moreira et al. made a comparison of the subdomain of the PDQ-39 between the mild and moderate stage PD patients and found that the worse QoL of the latter was related to the greater impairment in cognition (Moreira et al., 2017). Further analysis in our study revealed that the most affected subdomain of the PDQ-39 was bodily discomfort in both PD-NC and PD-MCI patients, while the cognition subdomain scored higher than bodily discomfort and motor dysfunction in PDD patients. This suggests that cognition probably makes more contribution to the QoL when PD patients develop dementia.

PD is characterized by motor function deficits, mainly manifested as rigidity, bradykinesia, and resting tremor (Kalia and Lang, 2015). It was reported that motor dysfunction was directly correlated with poor QoL in PD (Hinnell et al., 2012; Lawson et al., 2014, 2016). In support of this notion, we found that the UPDRS-III score, which measures motor function in PD, was an important determinant for QoL. However, a prospective study from Sweden found that the UPDRS-III score made no contribution to QoL of PD patients (Fereshtehnejad et al., 2015). The motor function impairment of the PD patients involved in the study (UPDRS-III score, 15.5 ± 9.2) was far milder than that of the patients in our study (UPDRS-III score, 34.93 ± 15.79), which may have led to contradictory results. In fact, compared to pure UPDRS-III score, physical function tests achieved a more systematic and comprehensive evaluation and showed greater values in predicting QoL in PD patients (Ellis et al., 2011). It was reported that the reduction of UPDRS-III score was positively correlated with better QoL in PD patients (Daniels et al., 2011), which suggests that the treatment of motor deficits makes great contributions to the improvement of QoL. Furthermore, we explored the impact of motor symptoms on QoL among PD patients with different cognitive statuses. The UPDRS-III score was shown to be the greatest contributor to QoL in PD-NC patients; however, while it is still a determinant, it makes the third strongest contribution to QoL in PD-MCI patients. The UPDRS-III score was not an important determinant for QoL in PDD patients, which is a somewhat unexpected result. We speculate that when PD patients develop dementia, they suffer from NMS that have a much greater effect on functional independence and QoL than motor symptoms. However, the results did not indicate that the treatment of motor deficits has no ameliorating effect on QoL in PDD patients. As this was a cross-sectional study, we could not exclude that interventions targeting motor symptoms might yield an improvement of QoL in PDD patients.

Different from the result that motor function showed the greatest effects on the QoL of PD-NC patients, depression, which was assessed by the GDS in this study, had the strongest impact on the QoL of PD-MCI and PDD patients. A series of studies reported that depression was the main and most frequently identified contributor of QoL for patients with PD (Winter et al., 2010, 2011; Hinnell et al., 2012; Lawson et al., 2014, 2016; Fereshtehnejad et al., 2015; van Uem et al., 2018; Kuhlman et al., 2019). Consistent with the other two studies (Kadastik-Eerme et al., 2015; Ophey et al., 2018), we also found that depression was the most critical determinant of QoL in all PD patients, emphasizing the impacts of depression. Nevertheless, clinicians frequently underestimate the importance of depression (Uitti, 2012). Depressive symptoms have often been ignored in PD patients (Shulman et al., 2002), and only a few depressed PD patients (less than 20%) underwent treatment for their depression (Weintraub et al., 2003). Therefore, doctors should place more emphasis on depression in PD patients, especially in those with cognitive dysfunction, for the purpose of maximizing the benefits on QoL.

Excessive daytime sleepiness is another common NMS in PD and increasing in prevalence with the advance of PD (Zhu et al., 2016). Our study indicated that EDS, evaluated by the ESS, was different among the three groups. In addition, consistent with previous studies that investigated the impacts of EDS on QoL (Kuhlman et al., 2019; Xiang et al., 2019; Yoo et al., 2019), we found that EDS was an important contributor to QoL in all PD patients. It was reported that EDS was independently correlated to cognitive impairment in PD (Marinus et al., 2018). Further analysis in our study found that EDS was an important determinant of QoL in PD-MCI patients, and tended to be a determinant of QoL in PDD patients, though with no significant difference (*P* = 0.0998), while it was not a determinant of QoL for PD-NC patients. In brief, our results suggest a unignorable role of EDS in QoL for PD patients with cognitive impairment. The use of dopamine agonists was considered an independent risk factor for EDS. In clinical practice, such knowledge should be taken into account before making appropriate treatment choices, particularly for those with cognitive dysfunction.

The strengths of the study include a relatively large number of enrolled PD patients and a full set of clinical and neuropsychological assessments. Nonetheless, there are still some limitations in our study. A major limitation of this study is the cross-sectional design, which could not analyze the longitudinal impacts of these factors on QoL and make causal inferences. Further longitudinal studies are required to explore whether QoL could be improved by clinical practice and interventions targeting these specific factors. Also, although all the patients were able to cooperate during the examination, partial data coming from questionnaires based on patient self-administered might be relatively less reliable for PDD patients. Finally, potential factors impacting QoL were analyzed by entering age, gender, education, disease duration, LED, UPDRS-III score, GDS score, SSST-12 score, ESS and RBDSQ score in multiple stepwise regression. Nonetheless, we cannot fully exclude the possibility of some other factors, such as falls, the use of anti-dementia and antidepressant drugs, were relevant to QoL. Future studies with information on these potentially important factors will need to minimize potential biases.

## Conclusion

Taken together, the current study indicated that the most important independent determinant of QoL was the UPDRS III score in PD-NC patients. Depression imposed a greater impact on QoL than motor function when cognitive impairment occurred in PD. To improve QoL, clinicians might need to focus on specific factors based on cognitive status in patients with PD.

## Data Availability Statement

The data that support the findings of this study are available on request from the corresponding authors (tangyilin@fudan.edu.cn; wangjian336@hotmail.com).

## Ethics Statement

The studies involving human participants were reviewed and approved by the Human Studies Institutional Review Board, Huashan Hospital, Fudan University. The patients/participants provided their written informed consent to participate in this study.

## Author Contributions

YF performed the acquisition of data, analyzed and interpreted the data, prepared the figure and drafted the manuscript for intellectual content. XL performed the statistical analysis and contributed to the data collection. LH, YSh, BS, CC, and YSu contributed to the data collection. JW and YT designed and conceptualized this study, interpreted data, and revised the manuscript for intellectual content. All authors read and approved the final manuscript.

## Conflict of Interest

The authors declare that the research was conducted in the absence of any commercial or financial relationships that could be construed as a potential conflict of interest.

## Abbreviations

EDS: excessive daytime sleepiness
ESS: epworth sleepiness score
GDS: geriatric depression rating scale
LED: levodopa-equivalent daily dose
MMSE: mini mental state examination
NMS: non-motor symptoms
PD: Parkinson’s disease
PDD: Parkinson’s disease with dementia
PD-MCI: Parkinson’s disease with mild cognitive impairment
PD-NC: Parkinson’s disease with normal cognition
PDQ-39: the 39-item Parkinson’s Disease Questionnaire
QoL: quality of life
RBDSQ: rapid-eye-movement sleep behavior disorder screening questionnaire
SI: summary index
SSST-12: sniffin’ sticks screening 12 test
UPDRS-III: unified parkinson’s disease rating scale part III.
