# Supplementary material for: Determinants of Quality of Life According to Cognitive Status in Parkinson’s Disease
Source: Front Aging Neurosci. 2020 Aug 20;12:269. doi: 10.3389/fnagi.2020.00269 (PMC7468499; doi:10.3389/fnagi.2020.00269)
Supplement: Supplementary file 1 [file Table_1.DOCX]

**Supplementary table 1: Normative data and direction of the neuropsychological tests.**

| **Cognitive test** | **Min score** | **Max score** | **Normative data (1.5SD)** | | | **direction** |
| --- | --- | --- | --- | --- | --- | --- |
|  |  |  | **50-59** | **60-69** | **70-79** |  |
| **Executive function** | | | | | |  |
| CWT-C time (s) [1] | / | / | ≤106 | ≤111 | ≤130 | ↓ |
| CWT-C right [1] | 0 | 50 | ≥40 | ≥38 | ≥35 | ↑ |
| TMT-B (s) （Education ＜12y）[1] | / | / | ≤200 | ≤230 | ≤290 | ↓ |
| TMT-B (s) （Education ≥12y）[1] | / | / | ≤200 | ≤210 | ≤240 | ↓ |
| **Attention and working memory** | | | | | |  |
| SDMT [1] | 0 | 110 | ≥29 | | | ↑ |
| TMT-A (s)（Education ＜12y）[1] | / | / | ≤85 | | | ↓ |
| TMT-A (s)（Education ≥12y）[1] | / | / | ≤70 | | | ↓ |
| **Language** | | | | | |  |
| BNT [2] | 0 | 30 | ≥20 | | | ↑ |
| AFT [3] | 0 | / | ≥10 | | | ↑ |
| **Memory** | | | | | |  |
| AVLT-delay recall [4] | 0 | 12 | ≥4 | ≥3 | ≥2 | ↑ |
| AVLT-T [4] | 0 | 60 | ≥20 | ≥18 | ≥15 | ↑ |
| CFT-delay recall [5] | 0 | 36 | ≥9 | ≥8 | ≥6 | ↑ |
| **Visuospatial function** | | | | | |  |
| CFT [5] | 0 | 36 | ≥32 | ≥30 | ≥29 | ↑ |
| CDT [6] | 0 | 30 | ≥17 | | | ↑ |

AFT, Animal Fluency Test; AVLT, Auditory Verbal Learning Test; BNT, Boston Naming Test; CDT, Clock Drawing Test; CFT, the Rey-Osterrieth Complex Figure Test; CWT, Stroop Color-Word Test; SD, standard deviation; SDMT, Symbol Digit Modality Test; TMT, Trail Making Test

Note: The values of CWT-C time, TMT-A and TMT-B are the completion time for the task

↑higher values are better; ↓higher values are worse.

[1] Guo QH, Sun YM, Yuan J, et al. Application of eight executive tests in participants at Shanghai communities. Chin J Behav Med Sci 2007; 16:628–3.

[2] Guo QH, Hong Z, Shi WX, et al. Boston naming test using by Chinese elderly, patient with mild cognitive impairment and Alzheimer’s dementia. Chin Ment Health J 2006; 20:81–5.

[3] Zhao QH, Guo QH, Shi WX, et al. Category verbal fluency test in identification and differential diagnosis of dementia. Chin J Clin Psychol 2007; 3:241–5.

[4] Guo QH, Sun YM, Yu PM, et al. Norm of auditory verbal learning test in the normal aged in Chinese community. Chin J Clin Psychol 2007; 15:132–5.

[5] Guo QH, Zhao QH, Chen MR, et al. A comparison study of mild cognitive impairment with 3 memory tests among Chinese individuals. Alz Dis Assoc Dis 2009; 23:253–9.

[6] Guo QH, Fu JH, Yuan J, et al. A study of validity of a new scoring system of clock drawing test. Chin J Neurol 2008; 41:234–7.

**Supplementary table 2. Cognitive profiles of PD-NC, PD-MCI and PDD patients**

|  | Total  (N=600) | PD-NC  (N=185) | PD-MCI  (N=336) | PDD  (N=79) | *P* Value* | *P* Value  PD-NC vs PD-MCI | | *P* Value  PD-NC vs PDD | | *P* Value PD-MCI vs PDD | |
| --- | --- | --- | --- | --- | --- | --- | --- | --- | --- | --- | --- |
| **Global cognitive abilities** | | | | | |  |  | |  | |  |
| MMSE | 26.44±3.32 | 28.21±1.58 | 26.79±2.28 | 20.76±3.98 | <0.0001 | <0.0001 | | <0.0001 | | <0.0001 | |
| **Attention and working memory** | | | | | |  |  | |  | |  |
| SDMT | 28.42±15.34 | 41.1±9.73 | 24.1±13.39 | 17.11±14.89 | <0.0001 | <0.0001 | | <0.0001 | | 0.0004 | |
| TMT-A (s) | 84.24±50.93 | 56.17±14.15 | 92.79±47.31 | 121.15±84.33 | <0.0001 | <0.0001 | | <0.0001 | | 0.0636 | |
| **Executive function** | | | | | |  |  | |  | |  |
| CWT-C time (s) | 93.13±44.06 | 76±16.38 | 95.82±36.98 | 126.31±84 | <0.0001 | <0.0001 | | <0.0001 | | 0.0004 | |
| CWT-C right | 44.28±5.93 | 46.65±3.14 | 43.57±6.54 | 41.17±6.57 | <0.0001 | <0.0001 | | <0.0001 | | 0.0021 | |
| TMT-B (s) | 189.04±86.77 | 141.21±39.94 | 206.03±82.81 | 251.22±131.85 | <0.0001 | <0.0001 | | <0.0001 | | 0.0066 | |
| **Language** | | | | | |  |  | |  | |  |
| AFT | 14.51±4.81 | 17.25±4.46 | 13.8±4.34 | 11.13±4.3 | <0.0001^†^ | <0.0001 | | <0.0001 | | <0.0001 | |
| BNT | 21.58±4.68 | 24.58±3.1 | 20.39±4.65 | 19.61±4.62 | <0.0001 | <0.0001 | | <0.0001 | | 0.3850 | |
| **Memory** | | | | | |  |  | |  | |  |
| AVLT-delay recall | 3.62±2.53 | 5.3±2.04 | 3.15±2.3 | 1.68±2.25 | <0.0001 | <0.0001 | | <0.0001 | | <0.0001 | |
| AVLT-T | 20.96±9.35 | 27.23±7.92 | 19.03±8.13 | 14.49±9.35 | <0.0001 | <0.0001 | | <0.0001 | | <0.0001 | |
| CFT-delay recall | 11.71±7.45 | 17.24±6.14 | 9.58±6.32 | 7.19±7.19 | <0.0001 | <0.0001 | | <0.0001 | | 0.0010 | |
| **Visuospatial function** | | | | | |  |  | |  | |  |
| CFT | 28.18±9.35 | 33.79±1.86 | 26.24±9.82 | 22.83±11.55 | <0.0001 | <0.0001 | | <0.0001 | | 0.1068 | |
| CDT | 18.58±7.50 | 22.48±5.24 | 17.18±7.59 | 13.67±7.62 | <0.0001 | <0.0001 | | <0.0001 | | 0.0058 | |

AFT, Animal Fluency Test; AVLT, Auditory Verbal Learning Test; BNT, Boston Naming Test; CDT, Clock Drawing Test; CFT, the Rey-Osterrieth Complex Figure Test; CWT, Stroop Color-Word Test; MMSE, Mini Mental State Examination; PDD, Parkinson’s disease with dementia; PD-MCI, Parkinson’s disease with mild cognitive impairment; PD-NC, Parkinson’s disease with normal cognition; SDMT, Symbol Digit Modality Test; TMT, Trail Making Test

The data are presented as mean ± SD.

Note: *Comparison among the three groups with PD-NC, PD-MCI, and PDD.

^#^The categorical variables were compared among the three groups by Chi-squared test.

^†^The continuous variable of AFT was compared among the three groups by one-way ANOVA test.

The other continuous variables were compared among the three groups by Kruskal-Wallis test.
